# LitDB - Keeping Track of Research Papers From Your Institute Made Simple

**DOI:** 10.1186/s13029-017-0065-2

**Published:** 2017-03-21

**Authors:** Jörn Bethune, Lars Kraemer, Ingo Thomsen, Andreas Keller, David Ellinghaus, Andre Franke

**Affiliations:** 1 Institute of Clinical Molecular Biology, Christian-Albrechts-University of Kiel, Kiel, Germany; 2 Chair for Clinical Bioinformatics, Saarland University, Saarbrücken, Germany

**Keywords:** Management of project-related publication lists, Database, Web browser interface, Impact factor, Citations, Open source

## Abstract

**Background:**

In science peer-reviewed publications serve as an important indicator of scientific excellence and productivity. Therefore, every scientist and institution must carefully maintain and update records of their scientific publications. However, in most institutions and universities articles are often managed in a redundant file-based and non-central way. Whereas excellent reference management software packages such as Zotero, Endnote or Mendeley exist to manage bibliographies and references when writing scientific articles, we are not aware of any open source database solution keeping track of publication records from large scientific groups, entire institutions and/or universities.

**Results:**

We here describe LitDB, a novel open source literature database solution for easy maintenance of publication lists assigned to various topics. In the last 2 years more than 50 users have been using LitDB at our research institute. The LitDB system is accessed via a web browser. Publications can be uploaded through direct exports from reference manager libraries or by entering PubMed IDs. Single users or user groups can track their citation counts, h-index and impact factor statistics and gain insights into the publication records of other users. It offers various visualization functions like coauthor networks and provides ways to organize publications from dedicated projects and user groups. The latter is in particular beneficial to manage publication lists of large research groups and research initiatives through a “crowd-sourcing” effort.

**Conclusions:**

Keeping track of papers authored and published by a research group, institute or university is an important and non-trivial task. By using a centralized web-based platform for publication management such as LitDB the compilation of project- and group-related publication lists becomes easily manageable and it is less likely that papers are forgotten along the way.

## Background

Scientific publications play an important role in research. The publication record is often used as an objective measure to benchmark productivity and excellence of a scientist within a research community. But modern scientists rarely work alone. They are part of research groups, academic departments or research initiatives. On each of these levels it is necessary to maintain current lists of publications to acquire third-party funding and to present a profile to the public.

Funding applications are usually written by senior researchers who are managing several scientists with less experience. The senior researchers not only need to keep track of their own projects and publications, but also of all the work that is done by the junior researchers which can be a lot of projects running in parallel. Given the importance of publications in academia, it is undesirable to not know about the relevant publication of a paper from your own research institution.

**Fig. 1:**
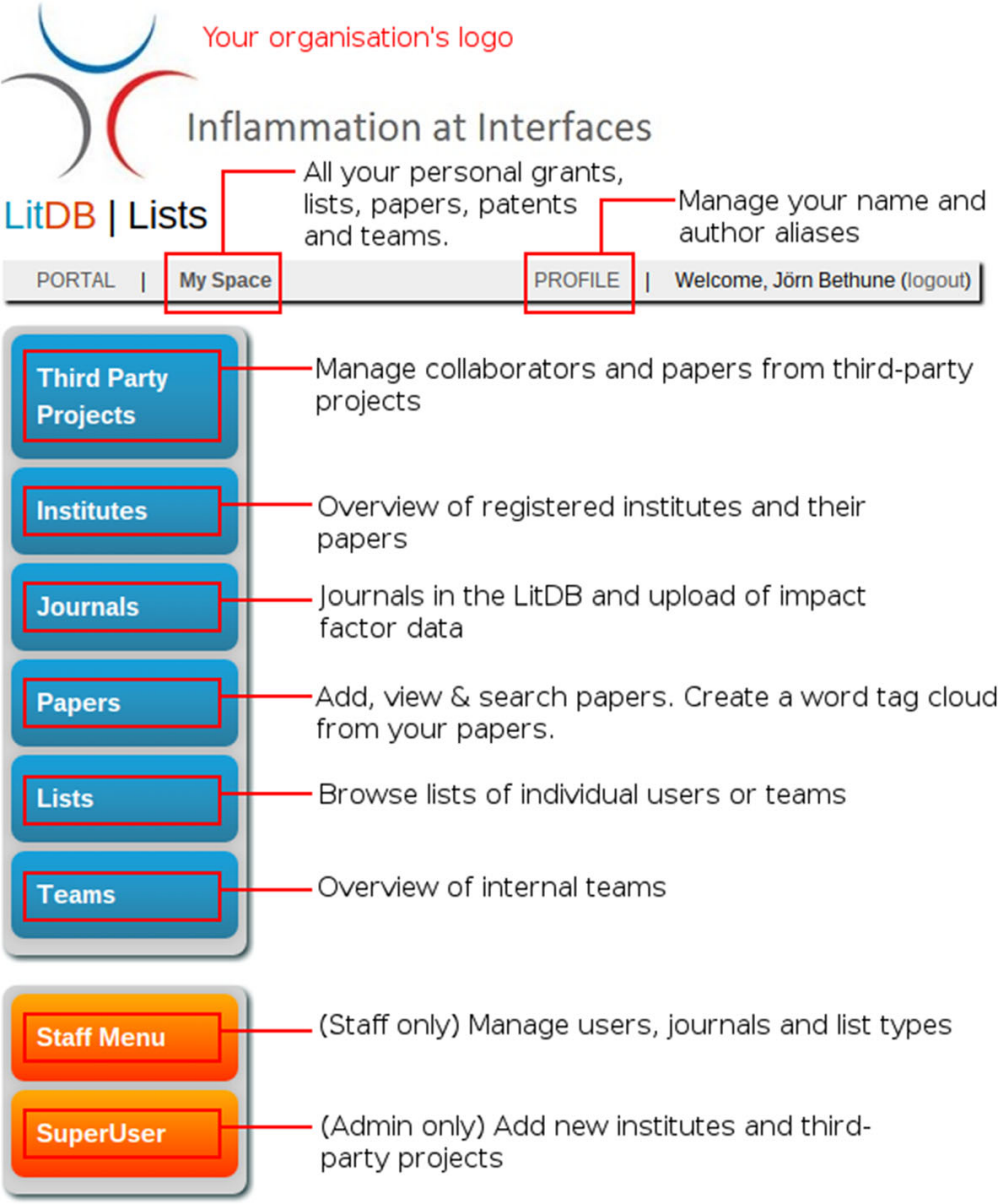
LitDB functionality overview. The figure depicts all options that are available to a super user. Normal users only see the blue menu options and staff users only see the staff menu. All entries in the LitDB that are related to the current user can be accessed under My Space. It is possible to configure LitDB so that a different logo is shown

Here we present a software-based solution to address this problem: We developed a literature management system called LitDB (Fig. 1). The LitDB software is a web-based application that allows researchers to store records of their own publications on a central server. This ensures that past publications are quickly discoverable for everyone in a research organization and that publication lists for project-related grant applications can easily be constructed.

The crucial idea behind LitDB is that all members of a research institute use the same database to keep track of their own publications and their own projects, so that other members of a research institute are aware of all publications that can be used in research grant applications.

## Implementation

LitDB is implemented in Python (version 2.7 [1]) with the Django web framework (version 1.8.7 [2]). It uses the PostgreSQL database [3] for data storage (via the python package psycopg2) and the D3.js [4] library for visualization of the co-author networks. LitDB has been tested on Microsoft Windows and Linux servers for the past 2 years. LitDB can make use of the open LDAP protocol (via the django-auth-ldap python package [5]) which is a standard for user management in most university’s computer systems, but is also capable of managing users as a stand-alone tool.

We provide a Docker [6] image that facilitates the deployment of the LitDB so that it is not necessary to deal with the software’s dependencies. For further technical information see the website of the LitDB: http://www.ikmb.uni-kiel.de/litdb.

## Results

**Fig. 2:**
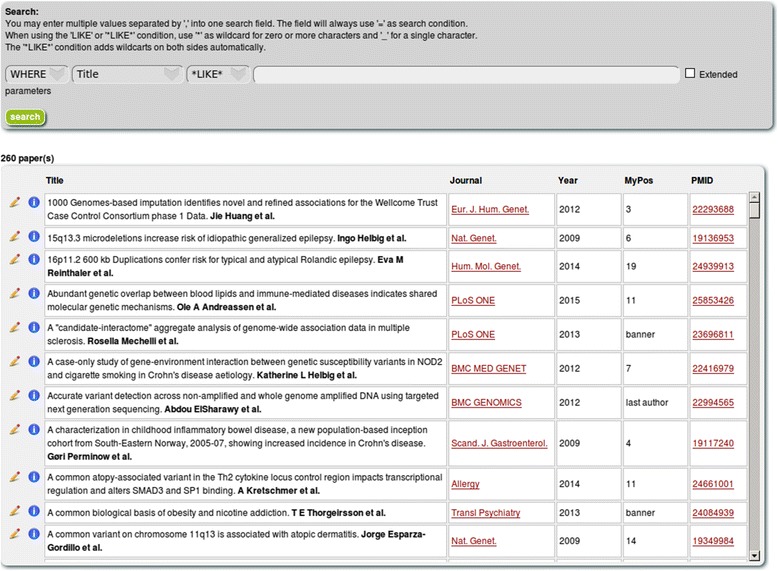
Listing of all papers from an author in the My Space section. With the help of the search form it is possible to search for specific papers. The overview table features the publication year and the author’s position for every publication. It is possible to edit entries to change incorrect information

The central feature of LitDB is the management of project-related papers. Papers can be submitted manually, but LitDB assists the user in the process. For instance, it is possible to only provide the PubMed ID of a paper and LitDB will gather all the remaining metadata by itself. It is also possible to add several papers at once by providing a list of PubMed IDs. If no PubMed ID is available (e.g. for book chapters), the users can still enter their papers by providing the details themselves. Every user can list his or her own papers in LitDB (Fig. 2). Further papers that the user might have coauthored are also suggested. However, these papers must have been previously submitted by another LitDB user. The user can then acknowledge or reject the suggested papers. In order for the LitDB to recognize the papers of the users, every user can provide aliases for his or her name. For instance, PubMed usually only saves the first letter of the first name of a person. But sometimes the complete first name is available for the search. Therefore, it makes sense to search for “John Doe” as well as for “J Doe”. This makes it also possible to take into account that people change names due to marriage, or to account for middle names or other circumstances. The problem of uniquely identifying scientists by their name(s) is not new. One popular solution to this problem is the use of unique IDs like ORCIDs [7]. The LitDB software also supports searching for papers by ORCID. Another useful feature is the possibility to categorize papers in the form of lists. When writing a grant application, it might be helpful to have a list that focuses on a specific scientific topic that is most relevant to the application that can be shared by several users. But other types of lists can also be created: A list of the top 5 publications of a user is a popular choice and shown on our institute’s webpage (with the help of an external script which queries the database). Lists can be created for single users but it is also possible to define projects in the LitDB and then all members of a project can create shared lists.

LitDB makes it possible to define working groups (teams), institutes and third-party projects so that all publications from a group of people can easily be obtained and employed for benchmarking, for example.

**Fig. 3:**
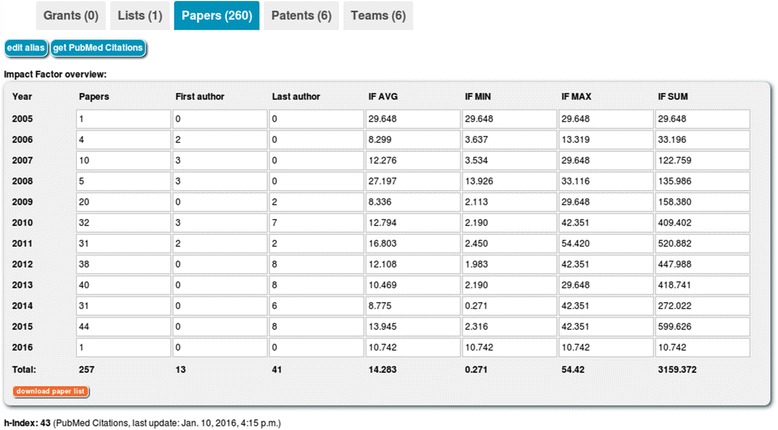
My Space section and paper overview. The user can create and manage grants, lists of papers, all of his papers, patents and teams in this section. In the current view, there is a table with yearly reports on the published papers. The number of papers, the number of first- and last-authorships as well as the average, minimum, maximum and total impact factors for every year are listed

As it was demanded by the users, we implemented in LitDB the additional feature to record research prizes, third-party grants and granted patents (Fig. 3), all of which are important benchmarking features as well and therefore often needed in grant applications and CVs.

LitDB also provides a table with statistics on the number of published papers and the impact factors for each year. This table can be used for writing annual reports and to compare the paper output of different years with each other.

## Alternatives

We have created LitDB because it provides a solution to a problem that we could not solve with other methods and non-commercial products. Most importantly, it provides a central hub for all authored papers for a group of researchers.

Often people use Microsoft Excel sheets or reference managers for keeping track of their publications. However, these tools are not well-integrated into larger context of a scientific institution. By using a central system, the scientists directly share their knowledge about their publications with their principal investigators, which leads to more consistent and sustainable archival of such information and makes it less likely that a publication is missed when writing a research grant. To our knowledge there are no other systems available to the general public that specialize on this task like LitDB does. It should be noted that LitDB does not try to replace reference managers. Its main purpose is to create central registry of all papers from a limited group of authors. Unlike popular reference managers like Endnote, Mendeley and Zotero, LitDB has been designed from the ground up for collaborative publication management.

LitDB also supports the calculation of impact factors for the papers in the system which is also helpful for raising funding for further research.

As the LitDB is a web-based application the researchers do not need to install any software beyond a web browser and everyone is able to work together with the same database, because there are no interoperability conflicts.

## Conclusions

Keeping track of the scientific publications of a larger group of researchers requires a lot of effort and diligence in order to not forget any publications that might be relevant for receiving further funding. The LitDB assists a research group with managing publication records based on the principle of “crowd-sourcing” to keep publication records up-to-date and complete.

These publication records are also very valuable for performing benchmarking calculations in combination with impact factors to measure and report scientific success over the years.

## Availability and requirements

**Project name**: LitDB

**Project home page**: http://www.ikmb.uni-kiel.de/litdb

**Operating system**: GNU/Linux

**Programming language**: Python (version 2)

**Other requirements**: A mod-wsgi compatible web server like Apache. Development libraries for libldap2, libsasl2 and libpq. The python packages django-auth-ldap and psycopg2. The Django web framework version 1.8.7

**Licence**: BSD License 2.0
